# Interventions for post-infectious irritable bowel syndrome: a systematic review of treatment efficacy

**DOI:** 10.1186/s40794-015-0002-9

**Published:** 2015-07-31

**Authors:** Emma Torbicki, Justin Oh, Sharmistha Mishra, Andrea V. Page, Andrea K. Boggild

**Affiliations:** 1 Faculty of Medicine, University of Toronto, Toronto, ON Canada; 2 Division of Infectious Diseases, Department of Medicine, University of Toronto, Toronto, ON Canada; 3 Division of Infectious Diseases, Department of Medicine, University Health Network / Mount Sinai Hospital, Toronto, ON Canada; 4 Tropical Disease Unit, Toronto General Hospital, Toronto, ON Canada; 5 Public Health Ontario Laboratories, Public Health Ontario, Toronto, ON Canada; 6 200 Elizabeth Street, 13EN-218, M5G 2C4 Toronto, ON Canada

**Keywords:** Chronic diarrhea, Gastroenteritis, Knowledge synthesis, Traveler’s diarrhea, Travel medicine

## Abstract

**Background:**

Post-infectious irritable bowel syndrome (PI-IBS) due to traveler’s diarrhea is the second most common illness seen in post-travel clinics, yet its optimal management remains unknown. We performed a systematic review to evaluate treatment efficacy in PI-IBS.

**Methods:**

We searched Medline, EMBASE, LILACS, CINAHL, CAB abstracts, and the Cochrane Library to February 3, 2014 for intervention studies of the pharmacologic and non-pharmacologic management of PI-IBS and examined the evidence according to a modified Grading of Recommendations Assessment, Development, and Evaluation (GRADE) scale.

**Results:**

Of 336 records, 9 studies were included. Eight studies of pharmacologic interventions examined 5 agents (mesalazine or mesalamine, ondansetron, prednisolone, cholestyramine, and metronidazole). One study examined the non-pharmacologic intervention of different infant nutritional formulas following acute gastroenteritis. The quality of the evidence to date was low, with small sample size (fewer than 50 participants) and short duration of follow-up. Overall, the efficacy of pharmacological treatment ranged from no benefit (ondansetron and prednisolone) to moderately beneficial (cholestyramine and metronidazole). The evidence for mesalazine was equivocal: one study showed benefit, two others showed none.

**Conclusions:**

Heterogeneity in outcome measures and low strength of evidence preclude recommendations on the optimal management of PI-IBS by a specific agent. More comparative intervention research into PI-IBS treatment is needed for consistent best practice in PI-IBS management. Clinicians may elect to pursue therapeutic trials of mesalazine, cholestyramine, or metronidazole in individual patients, but should be aware that data supporting the efficacy of these agents is limited.

**Electronic supplementary material:**

The online version of this article (doi:10.1186/s40794-015-0002-9) contains supplementary material, which is available to authorized users.

## Background

Traveler’s diarrhea (TD) is a frequent cause of illness in returning travelers, and it is most often attributable to bacterial causes [1–3]. It has been well documented that acute gastroenteritis and dysentery due to TD are associated with increased risk of developing post-infectious irritable bowel syndrome (PI-IBS) in both adults and children [4, 5]. Half of all travelers to the developing world experience diarrhea while abroad or upon return, and at least 10 % of those will develop PI-IBS [6]. During 2009–2011, PI-IBS was the second most common diagnosis among returning tourist travelers and the third most common diagnosis among all non-immigrant travelers evaluated at Canadian post-travel clinics [6]. PI-IBS results in abdominal discomfort, bloating, and diarrhea that persist for years despite clearance of the initial inciting pathogens [7, 8]. It has been postulated that PI-IBS may be caused by altered motility, increased intestinal permeability, and persistent intestinal inflammation, but the exact mechanisms of disease still need to be elucidated [7–10]. Diagnosis is typically made by documenting a history of gastroenteritis or dysentery and a new onset of IBS according to Rome criteria [7–10].

However, despite the relative frequency of PI-IBS, little synthesized evidence surrounding its management exists: there are no management guidelines or expert recommendations, and there is a distinct paucity of literature exploring treatment options. Therefore, there is a clear need for effective management guidelines [11, 12].

In order to determine the optimal evidence-based management of PI-IBS, we conducted a systematic review of the pharmacologic and non-pharmacologic interventions which have been evaluated to date for treatment of PI-IBS.

## Methods

### Search strategy

The search was conducted in two steps and in accordance with the Preferred Reporting Items for Systematic Reviews and Meta-Analyses (PRISMA) guidelines (PRISMA checklist included as Additional file 1: Appendix 1) [13]. First, we searched Medline, Excerpta Medica database (EMBASE), Literature in the Health Sciences in Latin America and the Caribbean (LILACS), Cumulative Index to Nursing and Allied Health Literature (CINAHL), Commonwealth Agricultural Bureaux (CAB) abstracts, and the Cochrane Library from inception to February 3, 2014 using combinations of the following search terms: “Post-infectious irritable bowel syndrome”, “post-infectious irritable bowel”, “post-infectious diarrhea”, “post-infectious constipation”, “post-infectious abdominal pain”, “post-infectious motility”, “post-infective irritable bowel”, and “post-diarrhea”. We restricted the search to English language papers and to studies conducted on humans. Additional file 1: Appendix 2 includes the Medline search. Second, we hand-searched reference lists and cited bibliographies from literature identified in the electronic database search but not meeting inclusion criteria for the systematic review for relevant papers, as well as the Public Health Agency of Canada/Committee to Advise on Tropical Medicine and Travel (http://www.phac-aspc.gc.ca/tmp-pmv/catmat-ccmtmv/index-eng.php) and Centers for Disease Control Websites (http://www.cdc.gov) for position and technical papers [14, 15].

### Inclusion criteria

We included all systematic reviews, randomized controlled trials (RCTs), clinical trials, cohort studies, observational studies, case–control studies, and larger case series (*N* > 5 participants) assessing or reporting the efficacy, safety, or tolerability of pharmacologic and non-pharmacologic interventions used in the management of post-infectious irritable bowel syndrome. For RCTs, we included placebo, comparator, or intervention trials and those with “no treatment” arms. Due to an anticipated paucity of high-quality studies, we included any trial fulfilling inclusion criteria, regardless of its definition of PI-IBS. We excluded case reports or small case series (*N* < =5 participants).

### Data extraction

We extracted all of the following possible outcomes, if and where reported: global symptom severity (efficacy); severity of abdominal pain (efficacy); frequency of diarrhea (efficacy); stool form and consistency as defined by the Bristol stool classification scale [16] (efficacy); severity of bloating (efficacy); subjective and objective tolerability of the drug; subjective and objective tolerability of the non-pharmacologic intervention; cost of intervention; duration of symptoms (efficacy); functional disability (efficacy); and adverse outcomes (safety). Data including intervention details, number of men and women in each group, average participant age, comorbidities (if reported), initial infectious organism (if reported), and article descriptors (e.g. title, authors) were extracted by two reviewers (ET, JO) and verified by one reviewer (AB) and stored in an MS Access database.

Quality and Strength of the evidence: We evaluated the studies included using a modified version of Grading of Recommendations Assessment, Development, and Evaluation (GRADE) [17]. GRADE is a system of rating evidence for systematic reviews and guideline development, which rates evidence from low to high quality [17]. It defines criteria for rating the quality of evidence for an intervention on specific outcomes based on: limitations, inconsistency, indirectness, imprecision, and publication bias. The quality of evidence for each treatment was evaluated across these criteria as appropriate given the number of studies per treatment and outcome.

Synthesis of intervention efficacy: We summarized the evidence using descriptive measures for each intervention type/agent. Meta-analysis of treatment efficacy was planned if at least five efficacy measures for the same treatment were available.

## Results

**Fig. 1:**
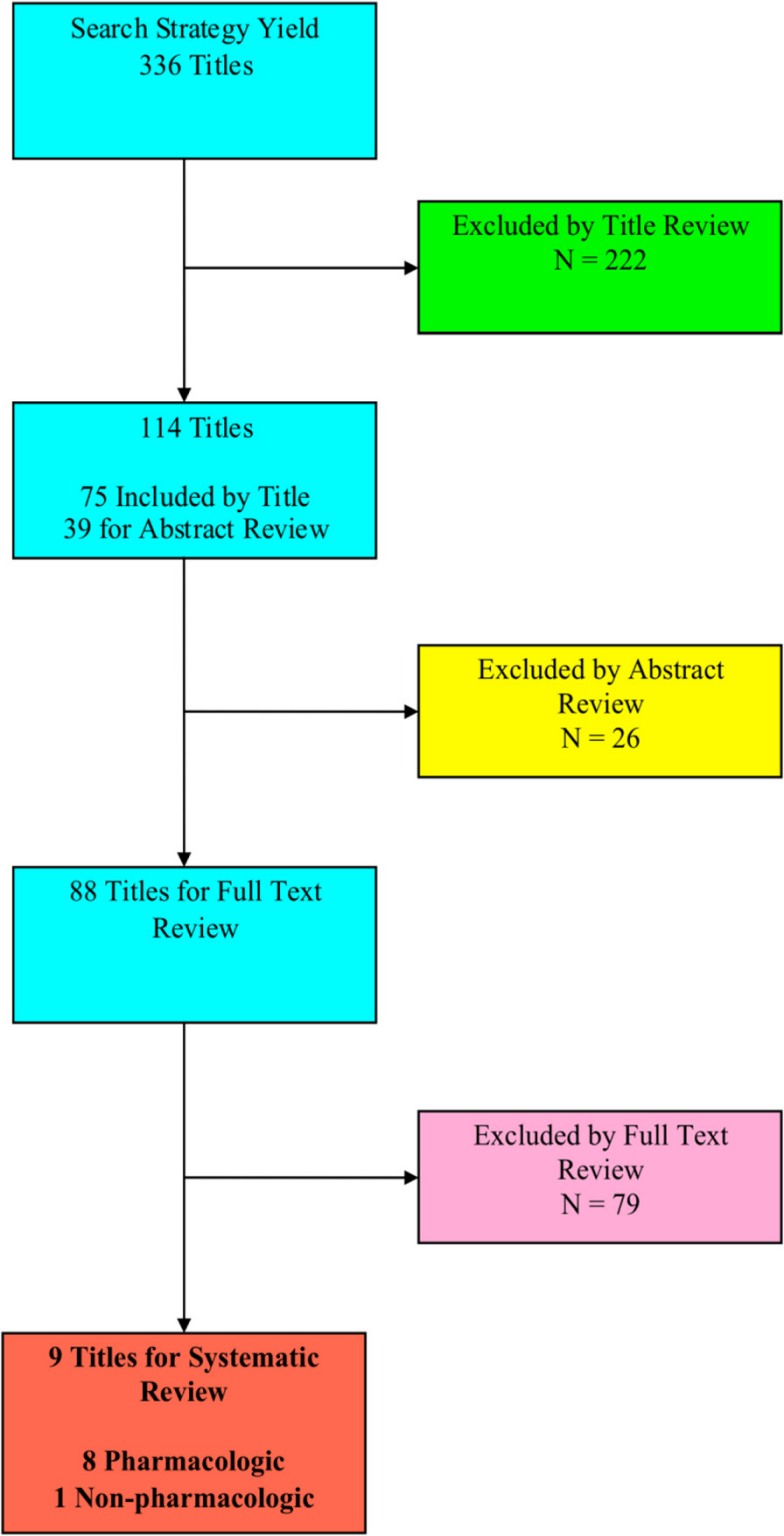
Flow diagram of article exclusions by stage of systematic review

The literature search returned 336 studies, of which 9 were included (Fig. 1): 8 evaluated pharmacologic interventions, and one evaluated a non-pharmacologic intervention. Of the 327 excluded articles, 222 were excluded at the title stage due to irrelevance to either IBS or PI-IBS. An additional 26 articles were excluded at the abstract stage due to irrelevance to either IBS or PI-IBS, despite titles suggestive of bowel syndrome-oriented primary research. Exclusion at the full text stage (*N* = 79) fell into 1 of 5 broad categories: review article with no primary data cited (*N* = 32); original research into the epidemiology, pathogenesis, diagnosis, or prognosis or PI-IBS, but without an evaluated treatment intervention (*N* = 12); therapeutic trial for IBS, without mention of PI-IBS (*N* = 9); therapeutic trial for prevention of traveler’s diarrhea (*N* = 8), and other (*N* = 18). Articles in the “other” category were excluded for various reasons such as publication only in a foreign language, publication in the form of an editorial or letter, or publication of a non-research article that had been updated subsequently by the same authors.

Study characteristics, quality of the evidence, and outcome measures of the included trials are shown in Table 1. Amongst the 8 studies examining pharmacological interventions, 5 different therapies were evaluated: mesalazine or mesalamine [18–20], ondansetron [21], prednisolone [22], cholestyramine [23, 24], and metronidazole [25]. We had an insufficient number of studies examining the same intervention on the same outcome measure to complete a full GRADE assessment. Thus, we used a modified GRADE rating consisting of only the limitations and indirectness categories (Table 2). The literature included in this study varied from low to moderate quality of evidence, due to reasons such as nature of study (*N* = 5), unclear allocation concealment and randomization method (*N* = 9) and differences in population of interest (*N* = 3, Table 1).

**Table 1:** Summary of interventions, study details, results and quality of evidence

Source	N (Intervention: Placebo)	Study Type	PI-IBS inclusion criteria	Intervention (Type, dose, length of intervention, frequency)	Duration of intervention	Outcome(s)	Quality of evidence (modified GRADE [17])
Bafutto *et al.,* 2011	61 (18:43)	cohort	Rome III with IBS-D, as reported by patient	- Mesalazine	30 days	- stool frequency decreased by 1.33 stools/day on average	Moderate
				- 30 days			The study is unclear as to how the randomization and allocation concealment were conducted.
				- 800 mg			
				- TID			
						- stool form and consistency improved by 2.17 based on the Bristol stool scale (16)	
Hanevik *et al.,* 2011	18 (11:7)	RCT	History of giardiasis 2 years prior and Rome II criteria	- Mesalazine	6 weeks	- no significant change in symptoms following mesalazine treatment	Low
				- 6 weeks			The study was a pilot study with small sample. It is unclear as to how to randomization and allocation concealment were conducted
				- 800 mg			
				- BID			
Tuteja *et al.,* 2012	20 (10:10)	RCT	Onset of IBS symptoms in previously asymptomatic individuals after acute gastroenteritis characterized by two or more of: diarrhea, vomiting, fever, as reported by the patient	- Mesalamine	12 weeks	- no significant change in symptoms following mesalazine treatment	Low
				- 12 weeks			Insufficient sample size
				- 1.6 g			
				- BID			
Dizdar *et al.,* 2007	34 (15:39)	RCT	Persisting abdominal symptoms 12 months after *Giardia* infection and Rome II criteria, as reported by Giardia outpatient clinic	- Ondansetron	2 days	- no significant improvement in symptoms after ondansetron treatment - Significant improvement in post-prandial nausea score before vs after ondanestron treatment (27.47 ± 21.89 vs 41.40 ± 23.04)	Moderate
				- 2 days			Small sample size
				- 8 mg			
				- QD			
Dunlop *et al.,* 2003	34 (20:14)	RCT	New bowel symptoms in a previously asymptomatic individuals immediately after an acute illness characterized by two or more of: diarrhea, fever, vomiting, positive stool culture, as recorded by the patient or gastroenterology clinic	- Prednisone	21 days	- No improvement in abdominal pain, diarrhea, frequency or urgency based on a gastrointestinal symptom rating scale	Moderate
				- 21 days			Small sample size
				- 30 mg			
				- QD			
Menon *et al.,* 2011	25 (15:10)	Retrospective	History of an acute gastroenteritis precipitating chronic diarrhea, as reported in clinic notes and hospital database	- Cholestyramine	Variable (1–15 years)	- decrease in diarrhea frequency of 5.9 stools/day	Low
				- 1–15 years			Retrospective study that only included people with bile acid malabsorption and infectious gastroenteritis.
				- 8.22 g			
				- QD			
Niaz *et al.,* 1997	16 (16:0)	Retrospective	History of an acute gastroenteritis precipitating chronic diarrhea defined as distinct change in bowel habit with 4–15 loose watery motions per day as reported by the patient	- Cholestyramine	2 weeks	- decreased stool frequency of 5.1 stools/day	Low
				- 2 weeks			Retrospective study that only included people with bile acid malabsorption and infectious gastroenteritis.
				- 2–16 g			
				- QD			
Thakur *et al.,* 2009	76 (17:59)	RCT	Rome II criteria and history of gastroenteritis or dysentery as reported by the patient	-Metronidazole	7 days	- improvement in pain, stool and total symptom scores at days 7 and 28	Low
							Study was not randomized, and both patient and physician were not blinded. 9 patients were lost to follow-up but it is unclear if they were included in analysis.
				- 4 weeks			
				- 400 mg			
				- TID			
						- stool symptoms continued to improve between day 7 and 28 even though patients were no longer taking the medication	
Lifshitz *et al.,* 1990	29 (10:19)	RCT	History of chronic diarrhea after an episode of gastroenteritis lasting more than 3 weeks as reported by Pediatric Gastroenterology unit	- Pregestimil	variable	- 9 out of 10 infants had improved clinical symptoms, shorter time to improvement	Moderate
				- 3 – 7 days			Population was limited to infants with lactose intolerance. Inclusion was based on chronic diarrhea, not on established criteria such as Rome criteria.
				- 1500 mL/kg			
				- Daily to provide 70 kcal/kg

**Table 2:** GRADE summary of findings table

Paper number	Limitations	Indirectness
1 (mesalazine)	AC: not discussed	Population: OK? IBS-D population selected based on ROME criteria but paper does not define how they separated IBS-D patients into post-infective and non-post-infective group.
	B: not discussed	
	FU: 0 pts lost to FU	
	OB: not a concern	
	L: none	
		Intervention: Good. Mesalazine is relevant intervention for our purpose.
	Question whether outcome measure scales were validated (abdominal pain and distention score)	
		Outcomes: Good. Outcomes are direct (e.g. abdominal pain, frequency of stool); concern regarding length of follow up (only 30 days)
		Indirect comparison: N/A
2 (ondansetron)	AC: randomized; method not discussed	Population: Good. Study population fulfilled ROME II criteria after Giardia infection.
	B: subjects and clinical investigators were blinded	
		Intervention: Ok. Ondansetron is relevant to our study, but the patients were fasted the day before and they were fed a specific meat soup.
	FU: 0 pts lost to FU	
	OB: not a concern	
	L: none	
		Outcome: Good. Direct outcomes such as abdominal pain/discomfort, nausea, fullness and satiety assessed; no information on chronicity; some surrogate measures (gastric emptying, max drinking capacity)
		Indirect comparison: N/A
3 (prednisolone)	AC: good “A designated phar- macist generated random sequences in blocks of six, five times.”	population: good, representative; Good. Is it problematic that some patients were recruited based on ROME I after gastroenteritis, and some on clinical diagnosis?
	B: patients and clinicians blinded; cell count was done blinded	
	FU: 5 pts lost to FU; analysis performed to take losses into account	intervention: good; Prednisone is relevant to our study.
	OB: not a concern	
		outcomes: surrogate measures (number of serotonin-containing enterochromaffin cells, number of lamina propria T cells) are the primary and secondary outcomes. But further direct outcomes were measured (pain, looseness, urgency and frequency) for up to 3 months; pts kept symptom diaries for 6 weeks of study
	L: none	
		Indirect comparison: N/A
4 (pregestimil)	AC: unclear	population: Ok. Limited study population to infants with lactose intolerance. Cannot be extended to adult population. Diagnosis by chronic diarrhea with gastroenteritis, so not certain if it would fulfill the IBS criteria.
	B: Good “The local physician handling the patients was not aware of the formula choice.”	
	NB: physician decided when and if change in feeding was needed – unblinding?	
		intervention: good, but specific to infants
	FU: 0 pts lost to FU	
	OB: not a concern	outcomes: good (direct outcome of days to improvement in diarrhea) but no information on longevity of effect.
	L: population age, population co-morbidities, outdated?	
		Indirect comparison: N/A
5 (cholestyramine)	AC: not done (retrospective)	population: Not good. Population mainly screened for bile acid malabsorption (75SeHCAT). Study population is not similar to our patients; some concerns about generalizability
	B: not blinded	
	FU: 7 pts stopped treatment, documented in study, rest of the study deals with only 18 pts who continued/responded	
	OB: not a concern	intervention: Not good. Cholestyramine would only be used in people who were diagnosed with bile malabsorption. Patients took cholestyramine in different doses and for different lengths of time. Patients were permitted to also take codeine phosphate and loperamide to alleviate symptoms; but this is not taken into consideration during the analysis.
	L: Stopping early for benefit: 6 pts who did not improve were not followed further Measures not validated?	
		outcomes: OK. Only mentions mean frequency of diarrhea as an outcome relevant to our study.
		Indirect comparison: N/A
6 (cholestyramine)	AC: not done (retrospective)	population: Not good. Population mainly screened for bile acid malabsorption (75SeHCAT). Study population is not similar to our patients; some concerns about generalizability
	B: not done	
	FU: 0 pts lost to FU	
	OB: not a concern	
	L: not a concern	
		intervention: Not good. Cholestyramine would only be used in people who were diagnosed with bile malabsorption. The doses of cholestyramine varied between patients.
		outcomes: Ok. Only mentions mean stool frequency as an outcome relevant to our study.
		Data only up to 2 weeks after start of treatment (longevity?)
		Indirect comparison: N/A
7 (metronidazole)	AC: not randomized, sorted based on disease type	population: good, representative (determined by ROME II and gastroenteritis).
	B: pts and clinicians not blinded	
	FU: 9 pts lost to FU; unclear if accounted in analysis	intervention: good
		outcomes: Good: validated questionnaire that assesses pain, stool frequency, consistency, etc. and patient follow up 3 weeks post study
	OB: validated symptom questionnaire used	
	L:	
		Indirect comparison: N/A
8 (mesalamine)	AC: good	population: good, representative (patients referred with clinical diagnosis of PI-IBS).
	B: participants and investigators blinded (head pharmacist allocated pts and meds)	
		intervention: good
	FU: 3 pts lost to FU; were accounted for in analysis	
		outcomes: Good: outcome measures of global improvements, mean change in symptoms, and quality of life.
	OB: no concerns (validated measures, clearly states primary and secondary outcome measures)	
	L:	Indirect comparison: N/A
9 (mesalazine)	AC: not discussed	population: Good: patients diagnosed with ROME II criteria after gastroenteritis.
	B: not discussed	
	FU: 2 pts withdrew due to AE	intervention: Good: mesalazine is relevant to our study.
	OB: not a concern	
	L: outcomes measured not clearly stated; “weekly symptom score/global improvement” used to measure outcomes, unsure of validity	
		outcomes: Ok. weekly Symptom scores and global improvement at the end of treatment measured, but do not describe what they are.
		Indirect comparison: N/A

Across the eight pharmacologic interventions, the efficacy of outcome ranged from no improvements to moderate reduction in bowel symptoms. Efficacy of mesalazine (*N* = 3) ranged from no improvement (bowel symptoms or quality of life, *N* = 2) to reducing daily stool frequency by 1.33 and a Bristol stool score [16] improvement by 2.17 (*N* = 1). Ondansteron (*N* = 1) did not provide any significant improvements to drinking capacity, gastric volume, and gastric emptying, but did improve the nausea score by 51 % compared to the placebo. Prednisolone (*N* = 1) did not show any improvements in abdominal pain, stool looseness, urgency, frequency, or global wellness score compared to the placebo. Treatment of post-infectious bile malabsorption with cholestyramine (*N* = 2) demonstrated 70.8–75.9 % decrease in stool frequency, from 7.2–7.83 stools per day to 1.89–2.1 stools per day. Metronidazole (*N* = 1) resulted in improvement in stool scores (urgency and frequency), pain response, and total symptom score.

### Mesalazine [18–20]

Bafutto, *et al.* [18] conducted a non-randomized interventional trial that compared the treatment response of PI-IBS patients and non-infective IBS patients, both diagnosed based on Rome III criteria, after 30 days of mesalazine therapy. In the PI-IBS group, the average stool frequency score decreased significantly from 2.44 at baseline to 1.11 on the 30th day after therapy (*P* < 0.0001). Stool form and consistency, as determined by the Bristol stool scale, improved significantly from 3.28 at baseline to 1.11 on the 30th day (*P* < 0.0001). Significant improvements in the same measures were also noted in the non-infective IBS group following mesalazine treatment.

The other two studies [19, 20] showed no significant change in symptoms following mesalazine treatment. Hanevik *et al.* [19] conducted an open pilot study of 18 patients with PI-IBS who were randomized to either mesalazine treatment or a non-treatment control group. There were no significant improvements in symptom scores in either group. Similarly, Tuteja *et al.* [20] conducted a pilot study with 17 patients with PI-IBS who were randomized to mesalazine treatment or placebo and found that mesalazine did not improve global symptoms, abdominal pain, bloating, stool urgency, frequency, or consistency, or quality of life score. These studies had longer treatment periods than the study by Bafutto *et al.*: six weeks [19] and three months [20], respectively.

### Ondansetron [21]

In a double-blind, randomized controlled trial, Dizdar *et al.* [21] randomized fifteen patients with PI-IBS to placebo or ondansetron treatment. The subjects were given 1 dose of ondansetron or placebo, fasted overnight, given a second dose of ondansetron or placebo, and then fed a meat soup test meal (Toro clear meat soup; Rieber & Son A/S, Bergen, Norway) in order to assess the drinking capacity, gastric volume, and gastric emptying via ultrasound. Symptoms were measured at the maximal drinking capacity after the ingestion of the soup. There was no significant improvement in abdominal pain, nausea, fullness, satiety, drinking capacity, 3D gastric volumes, or gastric emptying after the ondansetron treatment. However, the postprandial nausea score was significantly lower in the group that received ondanesteron than in the placebo group (27.47 ± 21.89 vs 41.40 ± 23.04 respectively, *P* < 0.005).

### Prednisolone [22]

In a double-blind, randomized controlled study by Dunlop *et al*. [22], twenty-nine PI-IBS patients were randomized to placebo or prednisolone and treated for 6 weeks. This study used cell counts from rectal biopsies as the primary and secondary outcome measures, but also included symptom scores as outcome measures. There was a decrease in gastrointestinal symptoms, consisting of abdominal pain, fecal looseness, urgency, and frequency, after the prednisolone treatment (*P* = 0.01). However, this decrease was not significantly different from the symptom reduction observed in the placebo group (*P* < 0.02). There was no statistically significant improvement in depression, anxiety, or global well-being score in the prednisolone treatment group. Patients receiving placebo demonstrated significantly greater improvement in the constipation syndrome score (1.0 ± 0.33) as compared to those receiving prednisolone (−0.4 ± 0.55) (*P* = 0.035).

### Cholestyramine [23, 24]

Two retrospective studies of patients with post-infectious bile acid malabsorption, a subset of PI-IBS, were included. In both studies, patients with post-infective bile acid malabsorption were identified based on results of a 75SeHCAT bile acid retention scan (a clinical test used to diagnose bile malabsorption) and a positive stool culture or clinical record of severe infectious gastroenteritis. In both studies, patients were allowed to titrate their own dose of cholestyramine, so the dose between patients varied from 2–16 g/day. Both studies were small, containing no more than 25 participants each. Menon *et al.* [23] reported that out of 25 patients, 7 patients stopped cholestyramine treatment due to constipation or no improvement in symptoms. However, 18 patients had a significant decrease in the frequency of bowel movements from 7.83 before the cholestyramine treatment to 1.89 stools/day afterwards (*P* = 0.001). The 18 patients who showed initial response had a sustained response to cholestyramine for over a year. Niaz *et al*. [24] reported a decrease in stool frequency from 7.2 stools/day one week before cholestyramine treatment to 2.1 stools/day after two weeks of cholestyramine treatment (*P* < 0.001).

### Metronidazole [25]

Thakur *et al.* [25] recruited 17 patients with PI-IBS, 24 patients with IBS-constipation (IBS-c) subtype, and 35 patients with IBS-diarrhea (IBS-d) subtype based on Rome II criteria and a history of acute gastroenteritis. The participants took metronidazole orally for seven days, and then returned on day twenty-eight for reassessment. There was a significant improvement in pain, modified stool score (stool frequency and consistency), and total symptom scores at days 7 and 28 compared to baseline. Stool symptoms continued to improve between day 7 and 28 in the PI-IBS group even though patients were no longer taking the medication. However, these improvements were not quantified in the manuscript. Both IBS-d and PI-IBS groups showed improved total symptom score and pain response compared to IBS-c (*P* < 0.05). In terms of stool symptoms, both the IBS-c and IBS-d groups improved in the first seven days, but unlike the PI-IBS group, these improvements were not sustained to 28 days.

### Infant formula [26]

The sole trial of non-pharmacologic interventions for PI-IBS investigated the response to three different dietary treatments in lactose intolerant infants with chronic post-infectious diarrhea [26]. This was a randomized intervention trial with no control arm, in which 29 infants were randomized to receive one of three dietary formulas (10 Pregestimil, 9 Portagen, and 10 soy). The three formulas differed primarily in carbohydrate, protein, and fat content, with Pregestimil having the highest carbohydrate content, but least amount of protein and fat. The average age of the infants was 4.8 months, and there were 17 males and 12 females. All infants were clinically diagnosed with acute gastroenteritis and diarrhea for more than 3 weeks, and the primary outcome of the study was improvement in diarrhea in 7 days. Infants receiving the Pregestimil formula demonstrated improved clinical symptoms, shorter time to improvement, and a lower rate of worsening diarrhea. Nine out of 10 infants had clinical improvement on Pregestimil (95 % CI: 0.71–1.09), 4 out of 9 infants improved on Portagen (95 % CI: 0.34–0.54), and only 1 out of 10 infants improved on soy (95 % CI: 0.04–0.16).

## Discussion

This systematic review of therapeutic interventions for PI-IBS demonstrated a lack of consistent, high-quality evidence to support an optimal management regimen for adults and children. Our search yielded 9 studies, of which one focused solely on various infant formulas and found that progestimil, a high carbohydrate and low protein and fat formula, lead to improvement in diarrhea in infants with PI-IBS and lactose intolerance. Eight others evaluated the effect of various medications on the symptoms of PI-IBS in adult patients. Treatment with either cholestyramine or metronidazole yielded moderate improvement in gastrointestinal symptoms in patients with PI-IBS. Treatment with ondansetron or prednisolone yielded no significant reduction in stool frequency, consistency, or pain, while treatment with mesalazine yielded variable results.

Very few randomized controlled trials have been conducted to evaluate interventions for PI-IBS. The available evidence is of low quality, and based on the frequency of PI-IBS in travelers returned from abroad, there is a need for higher quality research in this area. All of the studies available on management of PI-IBS deal with very small sample sizes (*N* < 50), which may not be large enough to capture the true effect of an intervention. Lack of homogeneity amongst reported outcome measures and the small number of studies hindered the pooling of results and meta-analysis. The follow-up periods in these studies were usually quite short, often less than 1 month. For a condition lasting an average of 2–3 years, this is insufficient to determine whether a treatment has had a lasting impact.

Current management practices for PI-IBS are largely based on the studies of patients with non-infectious IBS, and the treatments are mostly focused on symptom alleviation [8, 11]. For instance, patients are prescribed opiates for diarrhea, antispasmodics for abdominal discomfort, and tricyclic antidepressants for pain management [8]. In addition, soluble fiber and peppermint oil have been demonstrated to mitigate symptoms in randomized clinical trials of patients with IBS [27]. However, it must be noted that PI-IBS is a clinically distinct subgroup of IBS, with a specific inciting event and unique histologic and clinical features. PI-IBS necessarily follows an enteric infection, usually bacterial, and typically results in more serotonin-producing enterochromaffin cells (EC cells) and a higher number of mast cells in the gut [28, 29]. Patients with PI-IBS typically manifest more frequent diarrhea than constipation, and have fewer psychiatric comorbidities than do those with non-infectious IBS [28, 29]. These characteristics may have implications for treatment and its effect in patients with PI-IBS.

Depending on the severity of a patient’s PI-IBS symptoms, interventions such as mesalazine, cholestyramine, or metronidazole may be considered in therapeutic trials in individual patients. These interventions are based on biological plausibility, clinical research, and the current understanding of PI-IBS pathogenesis, which extends from in vitro and in vivo models of this syndrome. While some of the interventions described showed no benefit, including prednisolone and ondansetron, or had conflicting results in different studies (mesalazine), given the low quality of the available studies it is difficult to conclusively judge the effectiveness of these interventions. Patients should be informed of the available options, but also educated regarding the lack of evidence supporting those options and allowed to make their own treatment decisions. Patients should also be educated regarding the chronic nature of PI-IBS and encouraged to monitor their symptoms for patterns of worsening and improvement in order to identify potential triggers. Primary care physicians could aid patients through awareness of the risk factors for development of PI-IBS, and monitoring patients following episodes of acute gastroenteritis for development of PI-IBS. Counselling around the risk of PI-IBS in the pre-travel consultation setting may encourage greater attention to food and water precautions during travel, and through promotion of awareness, indirectly help to manage patient expectations should PI-IBS arise post-travel.

Limitations to our review include the use of keyword searches instead of medical sub-heading (MeSH) searches. Use of MeSH on databases like MEDLINE or EMBASE may be more sensitive than simple keyword searching. However, there was no MeSH term for PI-IBS, and an initial trial of MeSH related to IBS and management on MEDLINE and EMBASE yielded results that were neither sensitive nor specific. PI-IBS may not be included in the MeSH thesaurus because it is a relatively recently studied phenomenon, the term is conversational in that it has not been defined in the medical language, and PI-IBS is not yet considered sufficiently different from general IBS. Restriction of our search to English language articles may have compromised the sensitivity of our search strategy, and may have introduced bias to our results. We felt these risks would be low as PI-IBS is vastly over-represented in non-immigrant/non-‘visiting friends and relatives’ travelers traveling from developed to developing world settings [6], and due to the concentration of PI-IBS expertise in European, Australian, and North American centers. Other limitations include the lack of standardization of the definition of PI-IBS. Some of the included trials used Rome criteria to define PI-IBS, and not all provided evidence for exclusion of other causes of chronic gastrointestinal symptoms following travelers’ diarrhea, such as persistent infection, co-infection, or underlying gastrointestinal disease, all of which may have affected performance of the investigational therapeutic. Finally, heterogeneity in mechanism of action of the tested therapeutics and end-points made pooled analysis of the results impossible.

This knowledge synthesis has important consequences for individual patients, as well as for healthcare systems. Chronic diarrhea places a strain on healthcare resources as up to half of travelers returning from tropical and sub-tropical destinations develop infectious diarrhea [1–4, 6], and an estimated 10 % of those will go on to develop PI-IBS [30]. Despite the prevalence of PI-IBS, this systematic review found only 8 pharmacological and 1 non-pharmacological intervention trials specific to the management of PI-IBS. While interventions such as metronidazole, mesalazine, or cholestyramine may provide symptomatic relief, additional primary research into the pharmacologic management of PI-IBS is needed, as it is a clinically distinct subgroup of IBS with different underlying pathogenesis, and the evidence to support specific pharmacologic maneuvers, including those listed above, is extremely limited. Major research gaps in the management of PI-IBS exist, and priority should be given to evaluation of interventions that provide sustained symptomatic relief and target the underlying pathogenesis, thereby reducing morbidity, and improve the long-term prognosis of this disease. While non-pharmacologic interventions such as dietary restrictions, soluble fiber, and behavioural modifications are favored in the management of IBS, we found no evidence that these strategies extend to the PI-IBS population. A fulsome discussion of the risks, chronicity, and limited management options of PI-IBS should be entertained in the pre-travel setting so as to encourage adherence by travelers to food and water precautions while abroad, and prompt treatment of TD should it arise.

## Additional file

Additional file 1:
**Appendix 1.** PRISMA Guideline. **Appendix 2.** Medline Search.

## Abbreviations

BID: Twice daily
CAB: Commonwealth agricultural bureaux
CINAHL: Cumulative index to nursing and allied health literature
EC: Enterochromaffin
EMBASE: Excerpta medica database
GRADE: Grading of recommendations assessment, development, and evaluation
IBS: Irritable bowel syndrome
IBS-C: Irritable bowel syndrome with constipation
IBS-D: Irritable bowel syndrome with diarrhea
LILACS: Literature in the health sciences in latin America and the Caribbean
MeSH: Medical sub-heading
PI-IBS: Post-infectious irritable bowel syndrome
PRISMA: Preferred reporting items for systematic reviews and meta-analyses
QD: Once daily
RCT: Randomized controlled trial
TD: Traveller’s diarrhea
TID: Three times daily
